# Novel Applications of Artificial Intelligence in Cancer Research

**DOI:** 10.1177/15330338231195025

**Published:** 2023-08-13

**Authors:** Masayuki Tsuneki, Yudong Zhang

**Affiliations:** 1 Medical Research (Medmain Research), Medmain Inc, Fukuoka, Japan; 2 School of Computing and Mathematical Sciences, 4488 University of Leicester, Leicester, UK; 3 Department of Information Systems, Faculty of Computing and Information Technology, King Abdulaziz University, Jeddah, Saudi Arabia

**Keywords:** artificial intelligence, deep learning, machine learning, medical image analysis, diagnosis, treatment

## Abstract

Cutting-edge developments in machine learning and deep learning are improving all aspects of cancer research and treatment. Nowadays, the applications of artificial intelligence, machine learning, and deep learning to clinical aspects of cancer research have received more attention from scholars, with particular emphasis on diagnosis, prognosis, detection, and treatment.

The aim of this special collection titled “Novel Applications of Artificial Intelligence in Cancer Research” is to introduce the latest scientific research for the application of machine learning and deep learning (DL) approaches in medical image analysis (eg, computer-aided diagnosis, prognosis, detection, and treatment). There is no doubt that artificial intelligence is expected to be used in preventive medicine, cancer diagnostic support, personalized cancer treatment, and the discovery of new applications and techniques for cancer treatment. In this special collection, there are 11 original research articles as follows.

Wang *et al*^1^ obtained megakaryocyte count and classification for each sample by different methods (system-automated analysis, system-assisted analysis, and microscopic examination) to study the correlation between different counting and classification methods. The system showed outstanding performance in identifying megakaryocytes in bone marrow smears with high sensitivity (96.57%) and specificity (89.71%). The overall correlation between the different methods was confirmed by the high consistency (r ≥ 0.7218, *R*^2^ ≥ 0.5211) with microscopic examination in classifying megakaryocytes.

Zhang *et al*^2^ resented a machine learning model for an external/internal correlation prediction for lung tumor motion which is based on computed tomography radiomic features because it is necessary to determine the external and internal correlation individually before applying indirect tumor tracking. The model achieved the highest ROC-AUC at 0.946. Based on this study, radiomics is an effective tool for respiratory motion correlation prediction, which can extract tumor motion characteristics.

Tsuneki and Kanavati^3^ presented a DL model to classify gastric poorly differentiated adenocarcinoma in whole slide images (WSIs) of gastric endoscopic submucosal dissection (ESD) specimens. The trained model achieved ROC-AUC up to 0.975 in gastric ESD test sets for poorly differentiated adenocarcinoma. Based on this study, computational pathology applications which can assist pathologists in detecting and classifying gastric poorly differentiated adenocarcinoma from ESD WSIs would be of great benefit for routine histopathological diagnostic workflow.

Moshe *et al*^4^ demonstrated the use of TractSeg, which was proposed for automatic tract segmentation in healthy subjects, for corticospinal tract (CST) segmentation in a large cohort of patients with brain pathology and to evaluate its consistency in repeated measurements. This is a clinical application study for the existing model. Importantly, higher consistency between measurements was detected for the automatic segmentation, with between measurements correlations of volume = 0.92/0.65, MD = 0.94/0.75 for the automatic versus manual segmentation. This study revealed that TractSeg method was implemented for automatic segmentation in patients with brain pathologies, demonstrating superior consistency for the TractSeg-based CST segmentation.

Ren *et al*^5^ presented a Lung Cancer Data Augmented Ensemble (LCDAE) framework to solve the overfitting and lower performance problems in the lung cancer classification tasks. The LCDAE has 3 parts: The Lung Cancer Deep Convolutional GAN, which can synthesize images of lung cancer; A Data Augmented Ensemble model, which ensembles 6 fine-tuned transfer learning models for training, testing, and validating a lung cancer dataset; The third part is a Hybrid Data Augmentation, which combines all the data augmentation techniques in the LCDAE. By comparing existing state-of-the-art methods, the LCDAE obtains the best accuracy of 99.99%, the precision of 99.99%, and the F1-score of 99.99%.

Li *et al*^6^ compared the deep-learning-based auto-segmentation with atlas-based auto-segmentation for the superior constrictor, middle constrictor, inferior constrictor, and larynx. The mean Dice similarity coefficient values for the 4 structures were 0.67, 0.60, 0.65, and 0.84 for deep-learning-based auto-segmentation. In contrast, atlas-based auto-segmentation has Dice similarity coefficient results at 0.45, 0.36, 0.50, and 0.70, respectively. The mean 95th percentile of Hausdorff distance (cm) for the 4 structures were 0.41, 0.57, 0.59, and 0.54 for deep-learning-based auto-segmentation, but 0.78, 0.95, 0.96, and 1.23 for atlas-based auto-segmentation results, respectively.

Huang *et al*^7^ proposed a DL-based approach to predict intensity-modulated radiation therapy (IMRT) quality assurance (QA) gamma passing rates (GPRs) using delivery fluence informed by log files. The convolutional neural network (CNN)-based learning model was trained using delivery fluence as inputs and GPRs of 4 different criteria (3%/3 mm, 2%/3 mm, 3%/2 mm, and 2%/2 mm) as outputs. Model performance for both validation and test sets was assessed using mean absolute error, mean squared error (MSE), root MSE, Spearman rank correlation coefficients (Sr), and Determination coefficient (R^2^) between the measured and predicted GPR values. The CNN prediction model based on delivery fluence informed by log files could accurately predict IMRT QA passing rates for different gamma criteria. It could reduce the QA workload and improve efficiency in pretreatment QA. Their results suggest that the CNN prediction model based on delivery fluence informed by log files may be a promising tool for the gamma evaluation of IMRT QA.

Luan *et al*^8^ used CNN segmentation network to generate a series of contours, then use these contours as organ masks to erode and dilate to generate inner/outer shells for each 2D slice. Thirty-eight radiomics features were extracted from these 2 shells, using the inner/outer shells’ radiomics features ratios and DSCs as the input for 12 machine learning models. The authors used the DSC threshold adaptively classified the passing/un-passing slices. Through 2 different threshold analysis methods quantitatively, the authors evaluated the un-passing slices and obtained a series of location information of poor contours. From the isotropic experiments, almost all the predicted values were close to the labels. Through the anisotropic method, the authors obtained the contours’ location information by assessing the thresholds of the peak-to-peak and area-to-area ratios.

Zhu *et al*^9^ proposed a novel method (ReRNet) for classifying blood cells which could automatically classify blood cells and provide doctors with data as one of the criteria for diagnosing patients’ disease types and severity. In the ReRNet, ResNet50 is selected as the backbone, and 3 RNNs are used for classification. The ReRNet consists of 3 RNNs (SNN, dRVFL, and ELM) for classification. The model outputs were generated by the ensemble of results of 3 RNNs by majority voting, which provided improvement of the model robustness. Importantly, the ensemble of RNNs could improve classification performance. The ReRNet achieved the average accuracy at 99.97%, average sensitivity at 99.96%, average precision at 99.98%, and average F1 score at 99.97%. Therefore, the ReRNet is effective for blood cell classification.

Catteau *et al*^10^ analyzed a total of 151 biopsies of invasive breast. The Ki-67 index was evaluated by 2 pathologists with MIB-1 antibody as a global tumor index and also in a hotspot. These 2 areas were also analyzed by digital image analysis (DIA). For Ki-67 index assessment, in the global and hotspot tumor area, the concordances were very good between DIA and pathologists when DIA focused on the annotations made by pathologist (0.73 and 0.83, respectively). However, this was definitely not the case when DIA was not constrained within the pathologist's annotations and automatically established its global or hotspot area in the whole tissue sample (CCCs between 0.28 and 0.58).

Yu *et al*^11^ demonstrated the prediction of the voxel-based dose distribution for postoperative cervical cancer patients underwent volumetric modulated arc therapy using DL models. Authors adapted a 3D deep residual neural network (3DResUNet) and compared it with conventional 3DUNet. In comparison with the 3DUNet model, the predicted edge dose of the body in the 3DResUNet model is closer to the dose distribution of the treatment planning system. Authors showed feasibility and reasonable accuracy in the voxel-based dose prediction for postoperative cervical cancer underwent volumetric modulated arc therapy, which would be of clinical significance for the postoperative management of cervical cancer patients.

Teng *et al*^12^ collected US images of 148 cervical cancer patients. The 4 DL-based automatic segmentation models, namely U-net, context encoder network (CE-net), Resnet, and attention U-net were constructed to segment the tumor volumes automatically. Radiomics features were extracted and selected from manual and automatically segmented regions of interest to predict the LNM of these cervical cancer patients preoperatively. The reliability and reproducibility of radiomics features and the performances of prediction models were evaluated.

In the study of Ouyang *et al*,^13^ baseline MRI and clinical data were curated from patients with LARC and analyzed using logistic regression and DL methods to predict total neoadjuvant treatment (TNT) response retrospectively. The authors defined 2 groups of response to TNT as pathological complete response (pCR) versus non-pCR (Group 1), and high sensitivity [tumor regression grade (TRG) 0 and TRG 1] versus moderate sensitivity (TRG 2 or patients with TRG 3 and a reduction in tumor volume of at least 20% compared to baseline) versus low sensitivity (TRG 3 and a reduction in tumor volume < 20% compared to baseline) (Group 2).
